# Sleep Debt Elicits Negative Emotional Reaction through Diminished Amygdala-Anterior Cingulate Functional Connectivity

**DOI:** 10.1371/journal.pone.0056578

**Published:** 2013-02-13

**Authors:** Yuki Motomura, Shingo Kitamura, Kentaro Oba, Yuri Terasawa, Minori Enomoto, Yasuko Katayose, Akiko Hida, Yoshiya Moriguchi, Shigekazu Higuchi, Kazuo Mishima

**Affiliations:** 1 Department of Psychophysiology, National Institute of Mental Health, National Center of Neurology and Psychiatry, 4-1-1 Ogawa-Higashi, Kodaira, Tokyo, Japan; 2 Graduate School of Integrated Frontier Science, Kyushu University 6-10-1 Hakozaki, Higashi-ku, Fukuoka, Japan; 3 Faculty of Design, Kyushu University 4-9-1 Shiobaru, Minami-ku, Fukuoka, Japan; University of Pennsylvania School of Medicine, United States of America

## Abstract

**Objectives:**

Sleep debt reportedly increases emotional instability, such as anxiety and confusion, in addition to sleepiness and psychomotor impairment. However, the neural basis of emotional instability due to sleep debt has yet to be elucidated. This study investigated changes in emotional responses that are elicited by the simulation of short-term sleep loss and the brain regions responsible for these changes.

**Subjects and Methods:**

Fourteen healthy adult men aged 24.1±3.3 years (range, 20–32 years) participated in a within-subject crossover study consisting of 5-day sessions of both sleep debt (4 h for time in bed) and sleep control (8 h for time in bed). On the last day of each session, participants underwent polysomnography and completed the State-Trait Anxiety Inventory and Profile of Mood States questionnaires. In addition, functional magnetic resonance imaging was conducted while performing an emotional face viewing task.

**Results:**

Restricted sleep over the 5-day period increased the activity of the left amygdala in response to the facial expression of fear, whereas a happy facial expression did not change the activity. Restricted sleep also resulted in a significant decrease in the functional connectivity between the amygdala and the ventral anterior cingulate cortex (vACC) in proportion to the degree of sleep debt (as indicated by the percentage of slow wave sleep and δ wave power). This decrease was significantly correlated with activation of the left amygdala and deterioration of subjective mood state.

**Conclusion:**

The results of this study suggest that continuous and accumulating sleep debt that can be experienced in everyday life can downregulate the functional suppression of the amygdala by the vACC and consequently enhance the response of the amygdala to negative emotional stimuli. Such functional alteration in emotional control may, in part, be attributed to the neural basis of emotional instability during sleep debt.

## Introduction

Many people are now suffering from chronic sleep loss as a result of today's 24-h society, night-owl lifestyles, and prolonged work hours becoming a normal state of everyday life [1], [2], [3], [4], [5], [6]. Sleep loss causes day-time sleepiness and psychomotor impairment, and can result in human errors and accidents [7], [8], [9].

Acute sleep deprivation has been shown to augment physiological and psychological reactions to emotional stimuli. For example, compared with normal sleep conditions, overnight total sleep deprivation enhances sympathetic reactions to unpleasant stimuli, such as dilation of the pupils and increased heart rate and blood pressure [10], [11], declined task performance due to increased interference of working memory by unpleasant emotional stimuli [12], and increased changes in mood deterioration triggered even by weak emotional stressors [13]. According to functional brain imaging studies investigating the neural basis of emotional responses after acute sleep deprivation, unpleasant emotional stimuli increase the activity of the amygdala after overnight total sleep deprivation, suggesting a decline in functional connectivity between the amygdala and the medial prefrontal cortex (mPFC) which may reflect decreased inhibition by the frontal lobe [12], [14].

In addition, Swann et al. have shown that response time to subliminal priming is shortened after having short hours of sleep over a 2-day period [15]. Because subliminal visual information is transmitted to the amygdala without going through the visual cortex [16], [17], [18], [19], [20] and subliminal and supraliminal stimulation induce different responses in the amygdala [21], [22], it is possible that the transduction through subliminal signal pathways may also play an important role in changes in emotional responses to visual stimuli after sleep deprivation.

On the other hand, deteriorated mental and physical conditions due to sleep loss (partial sleep deprivation) are more likely to be caused by an accumulation of short sleep episodes over several days (sleep debt) than overnight total sleep deprivation [23]. Although sleep debt reportedly augments emotional instability (including anxiety and confusion), together with sleepiness, the feeling of fatigue, and deficits in psychomotor performance [24], [25], [26], [27], [28], the characteristics of emotional responses induced by sleep debt and the neural basis underlying such responses have not been studied extensively and therefore remain unclear.

In this study, we simulated continuous and accumulating sleep debt that can be experienced in everyday life (short hours of sleep over a 5-day period) to investigate changes in emotional responses caused by visual stimuli presented above and below the level of consciousness and the brain regions responsible for these changes.

## Materials and Methods

### Ethics Statement

This study was approved by the Ethics Committee of the National Center of Neurology and Psychiatry, Japan and was conducted in accordance with the Declaration of Helsinki.

### Participants

This study involved 14 healthy, right-handed adult men (mean ± SD age, 24.1±3.32 years) who provided written informed consent to participate. All participants were Japanese and native Japanese speakers. A sleep log and actigraph (Ambulatory Monitoring Inc., Ardsley, NY) were used to monitor the sleep schedule of participants during the observational period (a 2-week period prior to the study) and the following experimental period. Using Cole's algorithm with optimal parameters [29], sleep-onset time, wake time, and the amount of time awake in bed were calculated from the actigraph data and were compared with the sleep log to confirm the absence of irregular life patterns, such as working in shifts or staying up all night. Overnight polysomnography (PSG) was also conducted during the observational period to examine for sleep disorders.

Exclusion criteria were as follows: a mean bedtime or wakeup time during the observational period outside of the hours 23:00–02:00 and 07:00–10:00, respectively(including shift worker); some form of sleep disorder; serious physical complication; psychiatric disorder; ocular disease, including achromatopsia; taking medication or substances inveterately that might affect the experimental data (e.g., steroids and drugs that induce drowsiness such as hypnotics and anti-histamines); caffeine intake of over 200 mg per day, heavy smoker (stressed by a 5-day smoking cessation) implanted metal object such as a pacemaker; working shifts (engaged in shift work in the 4 weeks preceding the study); or travelling to a country with a 6-h time difference in the 3 months preceding the study.

### Sleep restriction protocol

**Figure 1:**
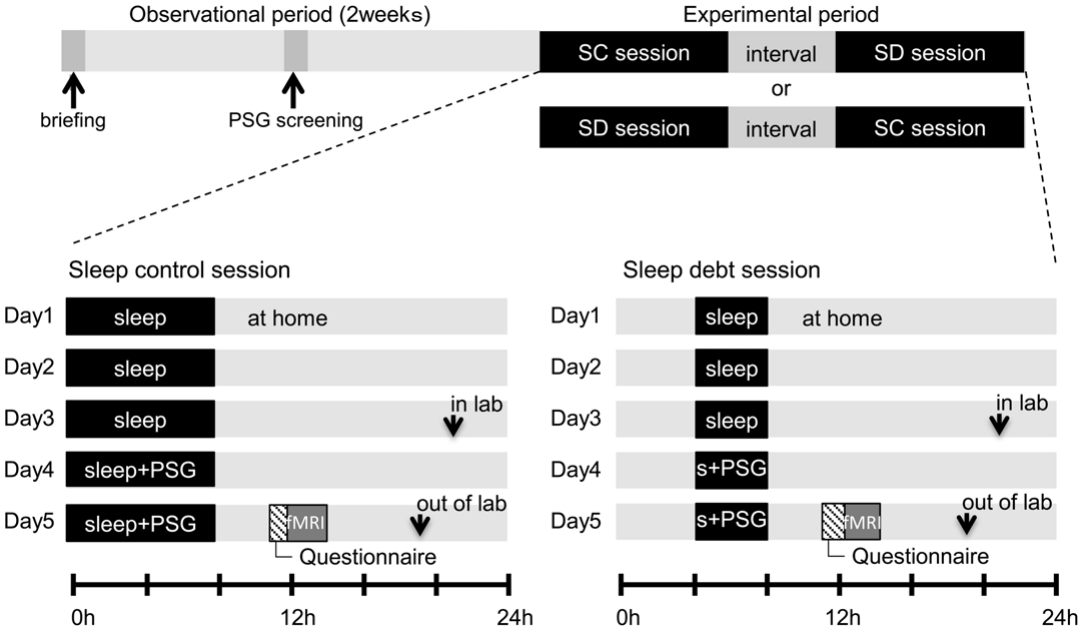
Experimental protocol. The study was conducted in a randomized crossover design, involving a sleep control (SC) and sleep debt (SD) session (for 5 days in each session) with a 2-week interval between the sessions. In the observational session before the experiment, participants visited the laboratory for a briefing session and gave their informed consent. One week later, participants came to the lab for PSG screening. One week after the PSG screening, the experimental sessions were started, with the order of the sessions counter-balanced across participants (i.e., SC-SD or SD-SC). Participants stayed at home on days 1–3 within each SC and SD session, according to the restrictive sleep-wake schedule that had been already instructed in the briefing (i.e., sleep time of 8 h for SC and 4 h for SD). Participants came to the lab on night 3 of the SC and SC sessions and spent the rest of the sessions (i.e., 2 days per session) in the sleep-lab with their sleep time controlled as instructed. On nights 3 and 4 in each session, participants underwent PSG measurement in the lab. On day 5, they completed questionnaires to check their mood state and sleepiness followed by fMRI scanning with an emotional task. SC, sleep control; SD, sleep debt; PSG, polysomnography; SSS, Stanford Sleepiness Scale; STAI, State-Trait Anxiety Inventory; POMS, Profile of Mood States.

Figure 1 shows the experimental protocol. All participants attended the briefing session concerning the experimental outline, underwent sleep electroencephalography (EEG) screening during the 2-week observational period, and participated in two 5-day experimental sessions. The number of hours in bed (i.e., after lights-out and during sleep) was 8 h/day in the sleep control (SC) session and 4 h in the sleep debt (SD) session. Both sessions were conducted as a crossover study with a 2-week interval between the sessions. During the interval, participants were asked to maintain a regular lifestyle without staying up all night or taking shift work.

In the SC session, based on the sleep log and actigram from the observation period, mean bedtime (23:00–02:00) for each individual was used as the start time for 8 h of sleep (wakeup time 07:00–10:00). In the SD session, bedtime started 4 h later (03:00–06:00) than that in the SC session and total hours in bed were 4 h (wakeup time 07:00–10:00).

In both sessions, participants stayed home for the first 3 days and then stayed in a laboratory room at the National Center of Neurology and Psychiatry for the next 2 days. To maintain the strict wakeup time at home, we sent a mail alert every 4 h, starting at the scheduled wakeup time until bedtime, and asked participants to answer the mail immediately. Participants were instructed by E-mail to refrain from caffeine and alcohol intake and smoking during the 5 days in which the sessions were held. In the laboratory, participants were under video camera surveillance, always assisted by a research attendant, and verbally awakened when in a drowsy state, such taking a nap or dozing off. During the wake period, participants were allowed to move freely around the laboratory, read and write, enjoy music and videos, play videogames, and engage in conversation with a researcher. Mineral water was always available, but the intake of caffeine and alcohol, smoking, and heavy exercise were restricted. Ambient temperature and humidity in the laboratory were maintained at 25±0.5°C and 50±5% RH, respectively.

### MRI and emotional face viewing task

Magnetic resonance imaging (MRI) was performed on day 5 of the sessions. Participants were first served the same breakfast (∼350-kcal sandwich) within 2 h of wakeup time before entering a room next to the MRI room 2–2.5 h after the wakeup time to answer a questionnaire about subjective sleepiness and mood. They underwent MRI 3–5 h after wakeup time.

**Figure 2:**
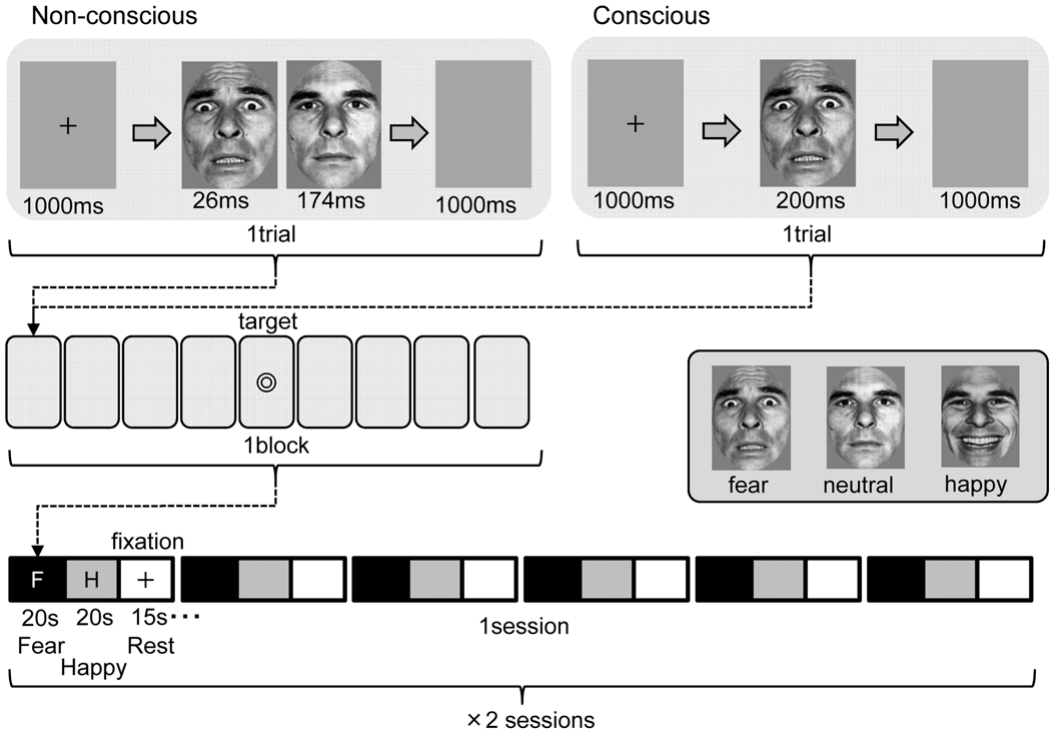
Design of emotional facial presentations. Facial pictures depicting fear or happy (i.e., emotional) or neutral expressions were used as the stimuli and were presented either non-consciously or consciously. In a non-conscious trial, an emotional image (either fear, happy, or neutral) was implicitly presented for 26 ms, followed by an explicit presentation for 174 ms of a neutral ‘masking’ face of the same identity as the preceding implicit emotional face (when the implicit face was neutral, the following explicit mask was of a different person of the same sex). Participants were required to press a button in response to each ‘target’ stimulus to keep themselves awake during the scanning.

During MRI, participants viewed faces with emotional expressions under two different conditions: (1) the conscious condition with a sufficient viewing time to allow supraliminal visual perception of an emotional facial expression and (2) the non-conscious condition with a brief viewing time to perform subliminal perception of an emotional facial expression followed by a neutral facial expression to mask the emotional facial image (Fig. 2).

We selected portraits of 16 individuals (4 Caucasian men, 4 Caucasian women, 4 Japanese men, and 4 Japanese women) from two standardized image sets ([30], [31] www.atr-p.com/face-db.html) and created 3 facial images per individual representing the categories of fear, happy, and neutral facial expressions by masking the hair and background (48 images in total).

(1) Under the conscious condition, a fixation image was presented for 1000 ms, followed by one of the three types of facial expressions for 200 ms and then a blank image for 1000 ms. (2) Under the non-conscious condition, after presenting a fixation image for 1000 ms, either (i) a neutral facial image was presented for 26 ms, followed by a neutral facial image of another person of the same sex for 174 ms and then a blank image for 1000 ms, or (ii) a happy (fear) facial image was presented for 26 ms, followed by a neutral facial image of the same person for 174 ms and then a blank image for 1000 ms.

In both the conscious and non-conscious presentations, therefore, one trial consisted of the presentations of a fixation image, a facial image in the conscious condition or two images in the non-conscious condition, and a blank image. Nine trials composed one block. Among each block of 9 trials, a target stimulus was presented randomly, to which participants were to respond by pressing a button in order to keep themselves awake and focused on the images. After completing each block, a fixation image was shown on the screen for 15 s (baseline). A total of 12 image presentation blocks (i.e., one session) were conducted under the non-conscious and conscious conditions (6 blocks each) with a 15-s baseline period provided every 2 blocks, and a total of 2 sessions were performed with a 2-min break between the sessions. The order of block presentation was counter-balanced between participants and sessions.

### Questionnaire

The Stanford Sleepiness Scale (SSS; [32]) was used to assess subjective sleepiness. Subjective mood was evaluated using state components of the State-Trait Anxiety Inventory (STAI-S; [33]), as well as the Profile of Mood States (POMS; [34]). During the SC and SD sessions, participants answered the questionnaire immediately prior to MRI.

### Polysomnography (PSG) and delta wave power analysis

On nights 4 and 5 of each session, PSG was performed and analyzed using the Neurofax EEG-1200 (Nihon Kohden Corporation, Tokyo, Japan) with Ag/AgCl electrodes. The system recorded an electrocardiogram (ECG), electrooculogram (EOG), electromyogram of the chin (Chin-EMG), and electroencephalogram (EEG) at C3, C4, O1, and O2 sites in line with the International 10–20 system using the mastoid processes as reference points. The sampling rate was 200 Hz, and the hardware bandpass filter was set at 0.5–35 Hz. EEG recordings from C3-A2 were used to perform visual classification of sleep stages in 30-s epochs in accordance with international sleep scoring parameters [35].

The PSG data obtained on night 4 was excluded from the analysis to eliminate the first night effect [36]. The following sleep parameters were calculated from the PSG data taken on night 5: total sleep time (TST), duration of each sleep stage, duration of slow wave sleep (Stage 3+4; SWS) during the 2-h period after bedtime, sleep latency (SL), sleep efficiency (SE), percentage of SWS spent in TST (%SWS), and percentage of rapid eye movement (REM) sleep (%REM).

Because sleep debt increases the duration of SWS and delta power during the initial stage of sleep [27], [37], [38], [39], [40], during the first 2 h after bedtime the amounts of SWS (SWS_2 h_) and δ power (δ_2 h_, 0.5–4 Hz) were used as objective indicators of sleep debt.

After visually excluding epochs containing body movement artifacts, all NREM sleep epochs (Stages 2–4) in the C3 EEG recording were analyzed using the fast Fourier transform (5.12-s hamming window, 2.5-s steps), and based on the power values obtained every 0.2 Hz, δ power (0.5–4 Hz) was calculated.

### fMRI acquisition

The Siemens Magnetom Verio 3T MRI system was used in the analysis. To obtain reference images for analysis, structural images (T1-weighted magnetization-prepared rapid gradient-echo (MPRAGE) images) were taken with the following sequence parameters: TR/TE = 1900/2.52 ms, voxel size = 1×1×1 mm, flip angle 9°, and field of view = 256×192 mm.

A single shot echo-planar imaging technique was used to obtain task-related functional MRI (fMRI) images. Settings were: TR/TE = 2500/25 ms, 30 axial slices, voxel size = 3×3×4 mm, 1-mm inter slice gap, flip angle 90°, matrix size = 64×64, and field of view = 192×192 mm. Of 137 scanning images obtained in each session, the first 5 images were excluded from analysis.

### fMRI data analysis

SPM8 (Wellcome Department of Imaging Neuroscience, http://www.fil.ion.ucl.ac.uk/spm/software/spm8/) was used in the analysis of functional brain imaging data. For each image, motion and slice timing correction as well as co-registration into an MPRAGE structural image was performed. MPRAGE imaging was carried out after PSG screening. The Montreal Neurological Institute (MNI) template was used for spatial normalization, and smoothing was performed using an 8-mm full width of half maximum Gaussian kernel. MRI time-series data that contained the three-dimensional blood oxygenation level dependent (BOLD) signals of each participant were analyzed using the first-level fixed effect model with general linear model (GLM) regression analysis. Using the canonical hemodynamic response model implemented in SPM8, a hypothetical hemodynamic time course corresponding to the stimulus presentations under each task condition was developed by convolving the canonical function. Thirteen hemodynamic models of time series corresponding to i) 6 conditions [3 categories of emotions (happy, fear, and neutral)×2 types of image presentation (conscious/non-conscious)], ii) target image presentation, and iii) 6 head motions as regressors were incorporated into the design matrix. Actual BOLD signals were analyzed voxel by voxel using the GLM, and the parameter estimate for each regressor was calculated. To subtract the low visual features, contrasts were created by subtracting the activity at the time of neutral facial image presentation from the activity at the time of emotional facial image presentation. Consequently, a total of 4 contrasts for a fear vs. neutral facial image and happy vs. neutral facial image under the conscious and non-conscious conditions were created.

To determine differences between the SC and SD sleep conditions, the value of the first-level contrast images in each SC and SD session were entered into a paired *t*-test implemented in SPM, with SC/SD as a within-subject factor.

Based on the hypothesis that sleep debt enhances the activity of the amygdala [14], we set the amygdala as the region of interest (ROI) and searched for the area where the activation was higher during the SD session than the SC session. Using the PickAtlas software (Wake Forest University (WFU), http://fmri.wfubmc.edu/downloads/WFU_PickAtlas_User_Manual.pdf) in the SPM Toolbox, masks for the amygdala on both sides were generated based on Anatomical Automatic Labeling (AAL), and each voxel in the mask was analyzed. Data were considered significant if p was less than 0.001 and the number of continuous voxels forming a cluster was greater than 5 within the amygdala ROIs. Furthermore, the significant cluster was corrected by family-wise error (FWE) correction within the amygdala ROIs (*p*<0.05, small volume correction [41]).

### Functional connectivity analysis

To reveal the functional connectivity related to enhanced amygdala activation during sleep debt, a cluster in the left amygdala (peak MNI coordinates x = −14, y = 4, z = −18, 8 voxels, see Results) that exhibited a significant difference in fear vs. neutral contrast between the SD and SC sessions was used as the seed region of the connectivity analysis. Based on a previous study hypothesizing that sleep debt weakens the functional connectivity between the amygdala and the mPFC adjacent to the ACC [14], we placed the ROI in the mPFC region for the functional connectivity analysis and searched for the area that exhibited greater connectivity with the seed region in the SC session than that in the SD session. Using the WFU PickAtlas software in the SPM Toolbox, a mask for the ACC/mPFC region was generated based on AAL (by combining ‘anterior cingulate’ and ‘medial frontal gyrus’), and each voxel in the mask was analyzed. The functional connectivity between the amygdala and the ACC/mPFC was analyzed using the CONN tool (Alfonso Nieto-Castanon, http://www.alfnie.com/software/conn) of SPM8.

Using GLM, voxels that were activated in relation to the BOLD signals in the seed region were extracted. Head motions and the hypothetical hemodynamic response to the main event (confounding effects of stimulus-locked transients [42]) and to the target were used as regressors, and the range of the bandpass-filter was set at 0.008–0.09 Hz. Connectivity contrasts thus created were used in first-level and second-level analyses, as in the analysis described earlier in the ‘fMRI data analysis’ section. Data were considered significant if *p* was less than 0.001 and the number of continuous voxels forming a cluster was greater than 5. Because of the larger size of the mask in the MPFC/ACC region, the small volume correction was not performed in functional connectivity analysis.

### Correlation analysis between mood/sleep changes and fMRI data

Differences in the values between sessions were calculated for subjective mood (scores from the STAI-S and POMS questionnaires), SWS_2 h_, and δ_2 h_. The contrast values between the sessions were also calculated for 1) amygdala activation and 2) the intensity of functional connectivity between the left amygdala and the ventral anterior cingulate cortex (FC_amg-vACC_) in the fear vs. neutral condition under conscious presentation. Correlations between these psychometric and imaging contrast values were analyzed.

Clusters used in the analysis were the left amygdala, which showed differential activation between the SD and SC sessions, and the vACC, which showed different degrees of functional connectivity with the amygdala, as the seed region, between the sessions. Marsbar software (Matthew Brett, http://marsbar.sourceforge.net/marsbar.pdf) was used to calculate the mean contrast values in a cluster.

### Statistics

The SPSS PASW Statistics 18 software package was used in statistical analysis. Differences in questionnaire scores, PSG data, and values of δ power between the SD and SC sessions were analyzed using the two-tailed *t-*test. Results are expressed as mean ± SD. Between-subjects test was performed by calculating Pearson's product moment correlation coefficient. Except for the analysis of functional brain activity, data were considered significant at *p*<0.05.

## Results

### Sleep time regulation

From the actigraph data, mean sleep time over the entire 5-day period in the SC and SD sessions was 8.09±0.35 h (8 h 5 min±21 min) and 4.60±0.54 h (4 h 36 min±32 min), with significantly fewer sleep hours (3.48±0.54 h, or 3 h 29 min±32 min) in the SD session [*t*(13) = 24.17, *p*<0.001].

### Sleepiness and mood states

Table 1 shows sleepiness and mood states associated with the SC and SD sessions. SSS and STAI-S scores for the SD session were significantly higher than those for the SC session, whereas no significant session-related differences were seen in POMS subscale scores.

**Table 1:** Subjective sleepiness and mood state scores for the sleep control (SC) and sleep debt (SD) sessions; *t* and *p*-values for SC vs. SD with the paired *t*-test.

	SC session	SD session	*t*	*p*
SSS	2.14 (0.66)	3.21 (1.05)	−3.51	<0.01
STAI-state	35.64 (6.21)	39.43 (4.86)	−2.74	<0.05
POMS Vigor	53.54 (10.02)	53.54 (11.57)	0.00	N.S.
POMS Depression	47.76 (9.00)	48.13 (8.71)	−0.40	N.S.
POMS Anger-Hostility	42.84 (4.52)	42.24 (6.18)	0.60	N.S.
POMS Fatigue	45.94 (6.61)	48.13 (7.48)	−1.30	N.S.
POMS Tension-Anxiety	46.37 (10.57)	47.05 (9.92)	−0.46	N.S.
POMS Confusion	48.12 (8.07)	49.94 (9.32)	−0.99	N.S.

Data are expressed as mean (standard deviation) values; SSS, Stanford Sleepiness Scale; STAI, State-Trait Anxiety Inventory.

POMS, Profile of Mood States.

Degrees of freedom (df) = 13.

### PSG/delta wave power data

Sleep parameters and the analysis results are shown in Table 2. Compared with the SC session, the duration of TST, Stage 1, Stage 2, and REM were significantly shorter in the SD session; however, no differences were observed with Stage 3+4 or SWS_2 h_. As a result, the SD session had significantly higher %SWS and SE and significantly shorter SL. In addition, δ_2 h_ for the SD session was significantly higher than that for the SC session.

**Table 2:** Values of spectral analysis and sleep variables for the sleep control (SC) and sleep debt (SD) sessions; *t* and *p*-values for SC vs. SD with the paired *t*-test.

	SC session	SD session	*t*	*p*
TST (min)	446.7 (22.5)	233.5 (7.5)	38.71	<0.001
Stage1 (min)	35.8 (20.0)	10.8 (8.2)	5.31	<0.001
Stage2 (min)	236.9 (35.3)	107.3 (28.9)	15.25	<0.001
Stage3 (min)	40.0 (11.3)	39.6 (15.4)	0.13	N.S.
Stage4 (min)	20.9 (25.8)	24.1 (25.8)	−1.52	N.S.
SWS (min)	60.8 (28.7)	63.7 (26.7)	−0.70	N.S.
REM (min)	113.1 (21.2)	51.7 (17.1)	11.09	<0.001
SWS_2 h_ (min)	37.5 (18.6)	41.4 (18.4)	−1.27	N.S.
Sleep latency (min)	19.3 (23.5)	3.3 (4.9)	2.90	<0.05
%SWS (%)	13.7 (6.7)	27.3 (11.6)	−7.21	<0.001
%REM (%)	25.4 (4.7)	22.2 (7.2)	1.80	N.S.
Sleep efficiency (%)	93.0 (4.7)	97.1 (3.1)	−3.46	<0.01
Delta_2 h_ (uV^2^/min)	1340 (275)	1495 (312)	−3.108	<0.01

Data are expressed as mean (standard deviation) values; SC, sleep control; SD, sleep debt; SWS, slow wave sleep.

SWS_2 h_, slow wave sleep of first 2 h from sleep onset; Delta_2 h_, Delta wave power (0.5–4 Hz) of first 2 h from sleep onset.

Degrees of freedom (df) = 13.

### Button Response

No significant session-related differences were seen in either the number or the mean time of responses (SC = 11.63±0.6, SD = 11.38±1.16, SC = 596.98±0.153.43, SD = 608.28±115.34, respectively).

### fMRI activation

**Figure 3:**
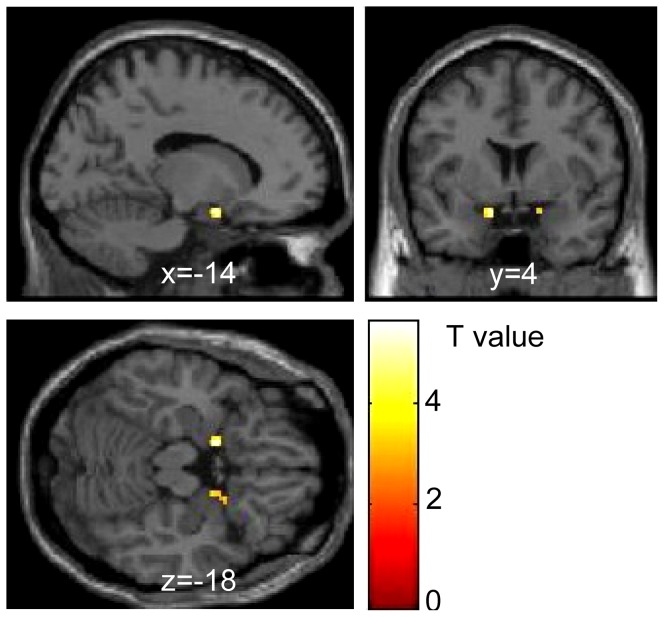
Difference in amygdala activation between the sleep control (SC) and sleep debt (SD) sessions. The map shows significantly greater activation in response to fearful face stimuli in the SD than SC session. Significant differences were seen in the left amygdala, peak MNI coordinate (*x*, *y*, *z*) = (−14, 4, −18) mm, *T*(13) = 5.60, *p* = .0001, *k* = 8 contiguous voxels. A similar trend was also observed in the right amygdala, (*x*, *y*, *z*) = (18, 2, −18) mm, *T*(13) = 3.41, *p* = .0005, *k* = 7. Significant clusters are rendered on a T1 anatomical referential image displayed in neurological convention, with the left side corresponding to the left hemisphere. The clusters shown are thresholded with a lenient alpha level (*p*<0.01, *k*>5) for visualization purposes. MNI, Montreal Neurological Institute template.

Comparison of fear vs. neutral contrasts for the conscious condition revealed significantly greater activation of the left amygdala in the SD session than in the SC session [peak MNI coordinates x = −14, y = 4, z = −18, *t*(13) = −5.60, FWE *p*<0.05 small volume correction] (Fig. 3; Table 3). Even though the activation of the right amygdala in the SD session was also higher than that in the SC session, it did not reach the significance level set for the analysis[peak MNI coordinates x = 18, y = 2, z = −18, *t*(13) = −3.41, *p* = 0.002]. With regard to happy vs. neutral contrasts, the amygdala showed no session-related differences in activation [left amygdala, x = −14, y = 4, z = −14, *t*(13) = −3.04; right amygdala, x = 16, y = 2, z = −16, *t*(13) = −1.90].

**Table 3:** Anatomical coordinates for regions of significant difference between the sleep control and sleep deprivation session compared with fear vs. neutral contrast.

Area		BA	MNI x	y	z	*t*	Z	*p*	Cluster k*
SD>SC Activity in amygdala mask									
Left	Amygdala	34	−14	4	−18	5.6	3.93	<0.001	9
SC>SD Functional connectivity with amygdala in ACC/MPFC mask									
Right	Anterior Cingulate	32	14	32	−4	4.77	3.56	<0.001	9

SC, sleep control session; SD, sleep debt session.

Cluster k* *p*<0.001 uncorrected threshold.

Degrees of freedom (df) = 13.

Under the non-conscious condition, no significant differences in amygdala activation between the sessions was observed with fear vs. neutral contrasts [left amygdala; x = −14, y = −8, z = −16, *t*(13) = −3.05; right amygdala, x = 24, y = −4, z = −12, *t*(13) = −3.08] or happy vs. neutral contrasts [left amygdala; x = −22, y = −8, z = −14, *t*(13) = −2.26; right amygdala, x = 26, y = −6, z = −16, *t*(13) = −2.60].

We performed the analysis of functional connectivity using only the conscious and fear conditions that showed differential activation between the SD and SC sessions.

### fMRI functional connectivity

**Figure 4:**
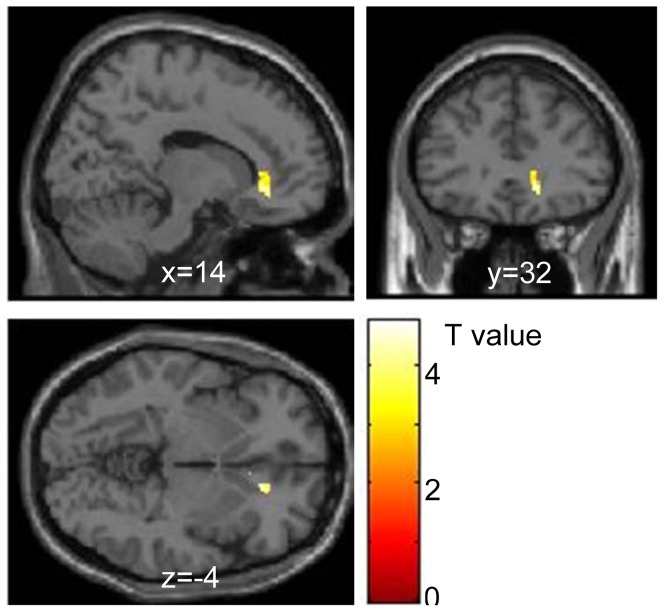
Difference in functional connectivity between sleep control (SC) and sleep debt (SD) sessions. The map shows greater functional connectivity between the left amygdala and other voxels in the brain in SC than SD session. Significant differences were found in the vACC, peak MNI coordinate (*x*, *y*, *z*) = (14, 32, −4) mm, *T*(13) = 4.77, *p* = .0001, *k* = 9 contiguous voxels. The significant cluster with a stronger connection with the left amygdala is rendered on a T1 anatomical referential image displayed in neurological convention, with the left side corresponding to the left hemisphere. The clusters shown are thresholded with a lenient alpha level (*p*<0.01, *k*>5) for visualization purposes. MNI, Montreal Neurological Institute template; vACC, ventral anterior cingulate cortex.

Comparative analysis of fear vs. neutral contrasts for the conscious condition revealed that, compared with the SC session, FC_amg-vACC_ was significantly diminished in the SD session [x = 14, y = 32, z = −4, *t*(13) = 4.77] (Fig. 4; Table 3).

**Figure 5:**
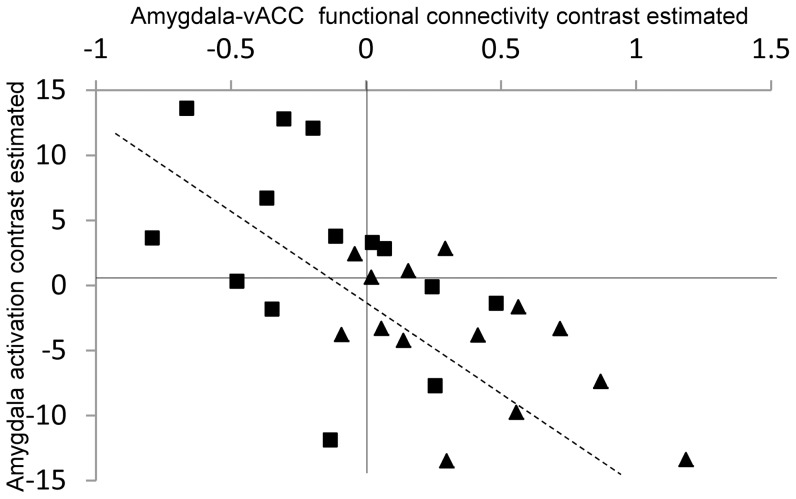
Correlation between amygdala activation and amygdala–vACC functional connectivity. Amygdala activation in response to fearful facial stimuli was negatively correlated with amygdala-vACC functional connectivity, *r*(13) = .64, *p* = .0001. The selected seed region within the amygdala was a cluster that showed greater functional connectivity with vACC in the SC than SD condition (*p*<0.001, uncorrected). Data from the SC and SD sessions were combined and plotted in one graph but differently colored; SD data in squares, SC in triangles. vACC, ventral anterior cingulate cortex; SC, sleep control condition; SD, sleep debt condition.

Analysis of all of the task results from the SD and SC sessions showed a significantly negative correlation between the activation of left amygdala and FC_amg-vACC_ [*r*(13) = 0.63, *p*<0.001] (Fig. 5).

### Correlations between mood/sleep changes and fMRI data

**Figure 6:**
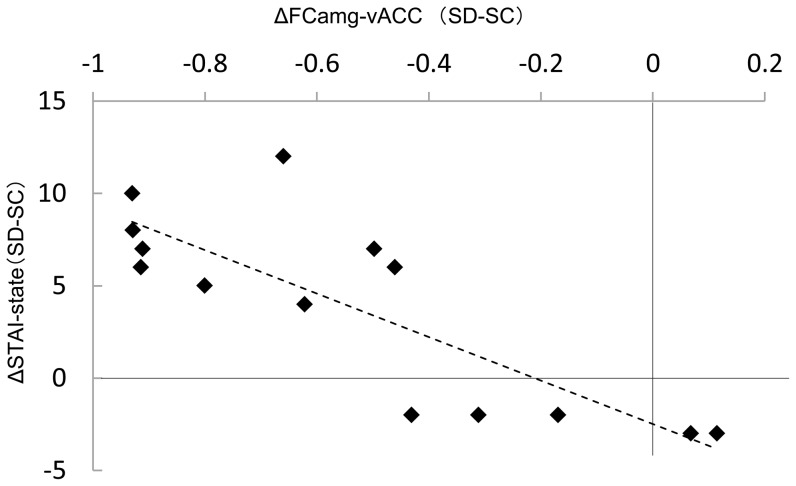
Correlation between the inter-session differences of amygdala–vACC functional connectivity and the inter-session differences STAI-state score. Inter-session differences between sleep control and sleep debt sessions of amygdala-vACC functional connectivity in response to fearful facial stimuli correlated negatively with inter-session differences of STAI-state score, *r*(13) = .82, *p* = .0001. Δvalue, inter-session difference between sleep control and sleep debt sessions for each value; vACC, ventral anterior cingulate cortex; SC, sleep control condition; SD, sleep debt condition; FC_amg-vACC_, functional connectivity between amygdala and ventral ACC; STAI, State-Trait Anxiety Inventory.

Sleep debt-related cross-correlation between left amygdala activation, FC_amg-vACC_, and changes in mood and sleep states are shown in Table 4. Changes of FC_amg-vACC_ between sessions (ΔFC_amg-vACC_) were negatively correlated with the changes of degree of sleep debt (ΔSWS_2 h_ and Δδ_2 h_) as well as mood changes (ΔSTAI-S (Fig. 6), ΔPOMS Tension-Anxiety, and ΔPOMS Confusion). On the other hand, no significant correlations were observed between Δamygdala activation and any of the parameters of the changes in mood or sleep states.

**Table 4:** Correlations between inter-session differences for fMRI data and questionnaire scores, and objective sleep debt Indices.

	ΔAmygdala activation	ΔFC_amg-vACC_
ΔSTAI-state	0.02	−0.82***
ΔPOMS Vigor	−0.22	0.49
ΔPOMS Depression	−0.20	−0.46
ΔPOMS Anger-Hostility	−0.38	0.11
ΔPOMS Fatigue	−0.27	−0.28
ΔPOMS Tension-Anxiety	0.13	−0.73**
ΔPOMS Confusion	0.02	−0.60*
ΔSSS	−0.41	0.01
ΔSWS_2 hrs_	0.36	−0.59*
ΔDelta_2 hrs_	0.03	−0.55*

Note. Δvalue, inter-session difference between sleep control and sleep debt sessions for each value; FC_amg-vACC_, functional connectivity between amygdala and ventral ACC; SSS, Stanford Sleepiness Scale; STAI, State-Trait Anxiety Inventory; POMS, Profile of Mood States; SWS_2 h_, slow wave sleep of first 2 h from sleep onset; Delta_2 h_, delta wave power of first 2 h from sleep onset;

* *p*<0.05,

** *p*<0.01,

*** *p*<0.001.

Degrees of freedom (df) = 13.

## Discussion

The results of this study revealed that sleep debt caused by having just a few hours of sleep for 5 days (3 h 29 min/day of sleep restriction compared with the SC session) increased the activity of the left amygdala in response to a fear facial image. In contrast, a happy facial image did not change the activity. Functional connectivity analysis demonstrated that the levels of FC_amg-vACC_ (left amygdala-vACC functional connectivity) were lower in participants with higher degrees of sleep debt (ΔSWS_2 h_ and Δδ_2 h_). The most important and novel finding in this paper is that declines in FC_amg-vACC_ were correlated with left amygdala activation and subjective mood deterioration (higher STAI-S and POMS scores). These findings strongly suggest that downregulation of the amygdala by the vACC and subsequent activation of the amygdala in response to negative emotional stimuli are involved in intensified physiological and psychological responses [10], [11], [12] and mood deterioration [13], [28] due to unpleasant emotional stimuli during sleep debt.

This interpretation is supported by a series of studies. The amygdala is thought to play an important role in the expression of negative emotions [43], [44], [45]. Facial expressions of fear were found to activate the amygdala even in healthy individuals with normal sleep [46], [47], and such activation is reportedly more prominent in individuals with depression and anxiety disorders [48], [49], [50]. Moreover, the amygdala has a strong functional and anatomical connection with the mPFC region [51], and the strength of this functional connection is correlated with the degree of subjective emotional suppression and reappraisal of negative affect. [12], [52], [53].

According to previous studies [12], [14], overnight total sleep deprivation diminishes the functional connectivity between the amygdala and the mPFC. In the present study, even a short-term, continuous and accumulating sleep debt that can occur in everyday life clearly resulted in reduced functional connectivity between the amygdala and the mPFC, and more specifically the vACC.

More importantly, subjective mood changes (increased anxiety) following short sleep were correlated with diminished FC_amg-vACC_, but not with the change in the activation in the amygdala (Δamygdala activation) itself. This may indicate that diminished synchronization between the amygdala and the vACC plays a more important role than the extent of the event-related local activation in the amygdala, for stabilizing increased anxiety evoked by an unpleasant emotional stimulus. Some models of amygdala functionality [53], [54] suggest that the magnitude of the local activity in the amygdala does not play a direct role in modulating the mood states of individuals, but the functional connectivity between the amygdala and the ventral mPFC correlates with STAI state score during resting state fMRI [55]. In our functional connectivity analysis, the main event-related hemodynamic response covaries with the seed-related (amygdala) activity in the GLM model of functional connectivity analysis; namely, main event-related immediate reactivity was regressed in our functional connectivity results (see Friston et al., 1997 [42] for the detailed process). Therefore, FC_amg-vACC_ in this study does not include simple event-related ‘co-activation’ between two regions, but finer event-unrelated synchronization between two regions beyond the local activities in two regions. This is why the local amygdala activity and the functional connectivity did not correlate with the psychological measurements in the same way; namely, the sustained anxiety (mood) state correlated with the FC_amg-vACC_, but not with the event-related local reactivity of the amygdala. This interpretation is supported by the diminished functional connectivity between the amygdala and the vACC or ventral mPFC, regardless of the amygdala's activity, in individuals with social anxiety disorder, in those with the s allele of the serotonin transporter gene and who thus have a high risk of depression, and in those with schizophrenia [56], [57], [58].

Although it has been shown that positive emotional stimuli also activate the amygdala [54], [55], happy facial expressions did not significantly alter amygdala activity during sleep debt in the present study. This suggests that functional changes in the amygdala and FC_amg-vACC_ during sleep debt become more apparent when negative emotional stimuli are presented. Overnight total sleep deprivation reportedly induces activation of the amygdala even in response to images associated with positive emotions [56]. It is reasonable to assume that this discrepancy is due to differences in the sleep conditions, such as total sleep deprivation in the former study and 5-day sleep restriction in the present one. It is possible that overnight sleep deprivation, more than accumulating sleep debt, affects the expression of positive emotion. In fact, overnight sleep deprivation elicits an antidepressant effect in patients with depression, as well as mood activation in healthy individuals [57], [58], [59], [60], [61]. Such effect and mood activation might be related to enhanced amygdala activation to positive emotional stimulus, as observed in a previous study. This notion is supported by the findings of a study in which antidepressant treatment enhanced amygdala activation in response to happy faces in patients with depression [62]. However, no similar phenomena have been reported in individuals with consecutive nights of sleep loss.

Unexpectedly, the presentation of emotional facial images under the non-conscious condition did not elicit changes in the amygdala activation after sleep restriction in the present study. A previous study showed that responses to masked priming are enhanced after a 2-day partial sleep deprivation [15]. Moreover, overnight total sleep deprivation strengthened the functional connectivity of amygdala with the sub-cortical region (midbrain) while diminishing the functional connectivity with the mPFC [14]. Based on these findings, we had expected to observe certain functional alteration in not only conscious processing through the cortical pathway, but also non-conscious processing through the subcortical pathway. However, no such alteration was observed in our participants. Any changes in non-conscious processing might be observable by adjusting the length of non-conscious image presentation, the intensity of the facial image stimuli, or increasing the sample size.

Interestingly, the decline in FC_amg-vACC_ was correlated with the increase in %SWS and δ wave power in the early period of the sleep, but not with subjective sleepiness. Because the increase in SWS and δ wave power is thought to be a sensitive indicator of the sleep homeostatic process [63], [64], it is possible that FC_amg-vACC_ is associated with the neural basis that enhances homeostatic sleep pressure following short hours of sleep. It is also thought that the increase in homeostatic sleep pressure is caused by the accumulation of sleep promoting substances in brain, such as prostaglandin D2 and adenosine [65], [66], [67], [68], [69]. For example, adenosine receptor antagonists have been shown to reduce sleep time by increasing wake hours and reduce slow wave activity during sleep [65], [66], [68]. Recent studies have hypothesized that adenosine, accumulated in the synaptic cleft because of consecutive synaptic transmission, binds to the A1 receptor to suppress synaptic transmission [70]. It is possible that synaptic suppression by a sleep-promoting substance is involved in the attenuation of FC_amg-vACC_ during sleep debt. Functional connectivity of the BOLD signal reportedly reflects a synaptic anatomical pathway [71]; therefore, it is expected that synaptic suppression by adenosine causes a decline in functional connectivity. This hypothesis is supported by a previous study which reported that a lack of sleep leads to attenuated resting state functional connectivity [72]. Moreover, a positron emission tomography (PET) study of human subjects revealed increased adenosine binding to the A1 receptor in regions including the vACC during sleep deprivation [73]. The use of PET with fMRI to elucidate the effects of sleep-promoting substances such as adenosine, including the effect of adenosine on the dynamics of neural activity in the brain, will likely elucidate the neural basis that alters the mechanism of emotion regulation.

Some limitations need to be taken into account when interpreting the present findings. First, participants in this study spent the first 3 days at home. Even though their sleep schedule was enforced by the use of the actigraph and mail alerts, their actual sleep time (8 h 5 min and 4 h 36 min in the SC and SD session, respectively) was longer than the scheduled sleep time (8 and 4 h in the SC and SD session, respectively). Despite the minor increase in sleep time, the SD session had a sleep loss of 3 h 30 min compared with the SC session. This level of sleep loss is rather frequently experienced in everyday life; however, the presence of sleep debt was confirmed on the last day of the session by the increase in %SWS and δ wave power. Because sleep requirements vary among individuals, the same sleep schedule may result in large individual differences in sleep debt [74]. In other words, even if everyone were to be placed strictly on the same sleep schedule, the effects of sleep debt on brain activity and subjective moods would vary widely among individuals. If sleep can be restricted based on individual sleep requirements, functional changes in the amygdala and FC_amg-vACC_ might be identified more accurately.

### Conclusion

The results of this study indicate that a short-term sleep loss, which is often experienced in everyday life, can aggravate subjective mood including anxiety, and the mechanism appears to involve functional alteration of the amygdala and FC_amg-vACC_. Long work hours, night-owl lifestyles, and an increase in shift work are the major contributors to sleep loss and thus the risk for depression [1], [2], [4], [6], [75], [76], [77], [78]. Therefore, ensuring adequate sleep is an important lifestyle factor that deserves more attention in terms of managing mental health, including depression.
