# Correction: Sleep Debt Elicits Negative Emotional Reaction through Diminished Amygdala-Anterior Cingulate Functional Connectivity

**DOI:** 10.1371/annotation/5970fff3-0a1c-4056-9396-408d76165c4d

**Published:** 2013-10-16

**Authors:** Yuki Motomura, Shingo Kitamura, Kentaro Oba, Yuri Terasawa, Minori Enomoto, Yasuko Katayose, Akiko Hida, Yoshiya Moriguchi, Shigekazu Higuchi, Kazuo Mishima

Department of Clinical Neuroimaging, Integrative Brain Imaging Center, National Center for Neurology and Psychiatry, 4-1-1 Ogawa-Higashi, Kodaira, Tokyo, Japan was missing from the affiliation list. The corrected affiliations are:

Yuki Motomura,1,2,3 Shingo Kitamura,1 Kentaro Oba,1,2 Yuri Terasawa,1,2 Minori Enomoto,1 Yasuko Katayose,1 Akiko Hida,1 Yoshiya Moriguchi,1,2 Shigekazu Higuchi,4 and Kazuo Mishima1,2,*

1 Department of Psychophysiology, National Institute of Mental Health, National Center of Neurology and Psychiatry, 4-1-1 Ogawa-Higashi, Kodaira, Tokyo, Japan

2 Department of Clinical Neuroimaging, Integrative Brain Imaging Center, National Center for Neurology and Psychiatry, 4-1-1 Ogawa-Higashi, Kodaira, Tokyo, Japan

3 Graduate School of Integrated Frontier Science, Kyushu University 6-10-1 Hakozaki, Higashi-ku, Fukuoka, Japan

4 Faculty of Design, Kyushu University 4-9-1 Shiobaru, Minami-ku, Fukuoka, Japan

University of Pennsylvania School of Medicine, United States of America 

